# The Prolactin Family of Hormones as Regulators of Maternal Mood and Behavior

**DOI:** 10.3389/fgwh.2021.767467

**Published:** 2021-12-01

**Authors:** Teodora Georgescu, Judith M. Swart, David R. Grattan, Rosemary S. E. Brown

**Affiliations:** ^1^Centre for Neuroendocrinology, University of Otago, Dunedin, New Zealand; ^2^Department of Anatomy, School of Biomedical Sciences, University of Otago, Dunedin, New Zealand; ^3^Department of Physiology, School of Biomedical Sciences, University of Otago, Dunedin, New Zealand; ^4^Maurice Wilkins Centre for Molecular Biodiscovery, Auckland, New Zealand

**Keywords:** prolactin, placental lactogen (PL), prolactin receptor, maternal behavior, maternal mood, neuroendocrinology, neural circuitry

## Abstract

Transition into motherhood involves profound physiological and behavioral adaptations that ensure the healthy development of offspring while maintaining maternal health. Dynamic fluctuations in key hormones during pregnancy and lactation induce these maternal adaptations by acting on neural circuits in the brain. Amongst these hormonal changes, lactogenic hormones (e.g., prolactin and its pregnancy-specific homolog, placental lactogen) are important regulators of these processes, and their receptors are located in key brain regions controlling emotional behaviors and maternal responses. With pregnancy and lactation also being associated with a marked elevation in the risk of developing mood disorders, it is important to understand how hormones are normally regulating mood and behavior during this time. It seems likely that pathological changes in mood could result from aberrant expression of these hormone-induced behavioral responses. Maternal mental health problems during pregnancy and the postpartum period represent a major barrier in developing healthy mother-infant interactions which are crucial for the child's development. In this review, we will examine the role lactogenic hormones play in driving a range of specific maternal behaviors, including motivation, protectiveness, and mother-pup interactions. Understanding how these hormones collectively act in a mother's brain to promote nurturing behaviors toward offspring will ultimately assist in treatment development and contribute to safeguarding a successful pregnancy.

## Introduction

The birth of immature offspring in mammalian species necessitates a period of intensive and prolonged caregiving by parents. In a number of species, including humans, and some primate and rodent species, fathers significantly contribute to the care-giving of new-born offspring. However, due to internal fertilization and the nutritional dependence of new-born offspring on lactation, mothers play an essential care-giving role in all mammalian species. Collectively termed “maternal behavior,” these care-giving behaviors by a mother have been defined as “responses or behaviors displayed by the female that specifically support the development and growth of her offspring” (1). As such, maternal behavior encompasses complex sets of behaviors that extend beyond the basic requirement for shelter and nourishment. The exact compilation of these behaviors and duration of their expression is determined in a species-specific manner. Nonetheless, these sets of behaviors can be broadly classified into either offspring-directed, to refer to direct interactions between mother and offspring, or offspring-related behaviors, which are not directed at the offspring but are required to ensure safety of young and to support the demands of lactation (1).

To understand the neurobiology underlying maternal mood and behavior in the postpartum period, it is important to note that maternal behavior represents a profound change in a female's behavior. Females in the maternal state will display a different set of behaviors, or perform these behaviors with a different intensity than is observed in non-pregnant females. For example, in many ungulate species, where females remain in close proximity to the herd, a parturient female will isolate herself in order to protect offspring either from the rest of the herd or from predators (2). In rodents, virgin rats will demonstrate maternal behaviors toward foster pups, but this process requires sensitization, involving repeated exposure to pups (3). In contrast, pregnant rats will spontaneously show maternal behavior 2 and 14 h prior to parturition (4). Although separation of learned vs. inherent maternal responses can be difficult to distinguish in primates, studies in non-human primate species, such as female macaques, have demonstrated that the frequency of infant-directed interactions specifically increases during pregnancy and lactation (5). The onset of enhanced maternal responses being so precisely timed with the birth of offspring, points to an important role for the hormones of pregnancy and lactation in promoting these behaviors. First described in 1972, a humoral basis for maternal behavior (6) has since been the subject of extensive research in a wide variety of mammalian and non-mammalian species. Extensively reviewed elsewhere, multiple hormonal signals contribute to the onset and maintenance of maternal behaviors, including estrogen, progesterone, oxytocin, and prolactin (1, 7–13). Rather than reviewing the role of all these hormones in-depth here, the aim of this review is to provide a focused examination of the literature on the role of the prolactin family of hormones across multiple facets of maternal behavior.

Although prolactin itself has been the focus of many studies, it is but one of a wider group of hormones classed as “lactogenic hormones” that act through a common receptor, the prolactin receptor (Prlr). Lactogenic hormones encompass a group of single peptide hormones of around 200 amino acids that stimulate milk production, and include prolactin, placental lactogens (or chorionic somatomammotropin in women), and in humans, also include growth hormone. In non-pregnant females, circulating prolactin is maintained at low basal levels through a negative feedback loop system. Secretion of placental lactogen or human chorionic somatomammotropin is initiated early in pregnancy with circulating levels increasing until parturition (10). The lactogenic composition during pregnancy is distinct in rodents, with the mating stimulus inducing twice-daily surges in prolactin secretion for the first 9 days of pregnancy (14). However, in the second half of pregnancy, placental lactogen I and II sequentially become the main source of circulating lactogenic hormones present in the blood (15, 16). The elevated placental lactogens act *via* Prlr in the hypothalamus to inhibit maternal prolactin secretion through negative feedback (17, 18). This negative feedback regulation disappears in late pregnancy, and suckling induces chronically high levels of prolactin secretion throughout lactation (19). When assessing a potential role for these hormones in influencing maternal behavior, it is important to consider the circulating levels of both placental lactogen (and growth hormone in women) and prolactin during pregnancy and lactation.

Recent studies have suggested that lactogenic hormones may have an important role in regulating postpartum mood in women. Prenatal depression in women is associated with low levels of placental lactogen (20), and postpartum depression has also been linked to low placental lactogen levels at term in mothers who give birth to female infants (21). Potentially one of the primary causes of low placental lactogen production in women is maternal obesity (22), a concurrent risk factor for developing postpartum depression and anxiety (23–25). This finding has been replicated in animal models, with mouse models of maternal obesity leading to suppressed placental lactogen production (26) and poor maternal behavior (27). Furthermore, in addition to effects on placental hormone production during pregnancy, maternal obesity in women is also associated with low prolactin secretion during lactation (28, 29). To date, very few studies have investigated the role of lactogens in regulating maternal mood and behavior in women, and our current understanding is derived from mechanistic studies in animal models. In particular, due to their small size, short reproductive life-cycle, and the development of transgenic tools, rodents have been invaluable in identifying how lactogenic hormones influence maternal behavior. The aim of this review is to incorporate what has been learnt from these animal models about the role of lactogenic hormones in both offspring-directed and offspring-related maternal behaviors, and to consider how this might impact on mood in women.

## Lactogenic Signaling in the Brain

The Prlr is expressed by a wide range of tissues throughout the body and also in the brain (30–33). At 23 kDa in size, lactogens are too large to cross the blood-brain barrier to exert central actions in the absence of a transporter. Peripherally-derived prolactin enters the brain through a saturable carrier-mediated transport system (34, 35), with levels of prolactin in the cerebrospinal fluid paralleling changes in peripherally circulating prolactin (36, 37). While initially thought to be dependent on Prlr expression within the choroid plexus of the brain's ventricular system, recent studies have demonstrated that prolactin transport does not rely on the Prlr, but rather on a currently unidentified transport molecule (35). In addition to peripherally-derived lactogens acting in the brain, recent data also suggests that prolactin itself can be synthesized and released into the cerebrospinal fluid by epithelial cells in the choroid plexus (38).

**Figure 1:**
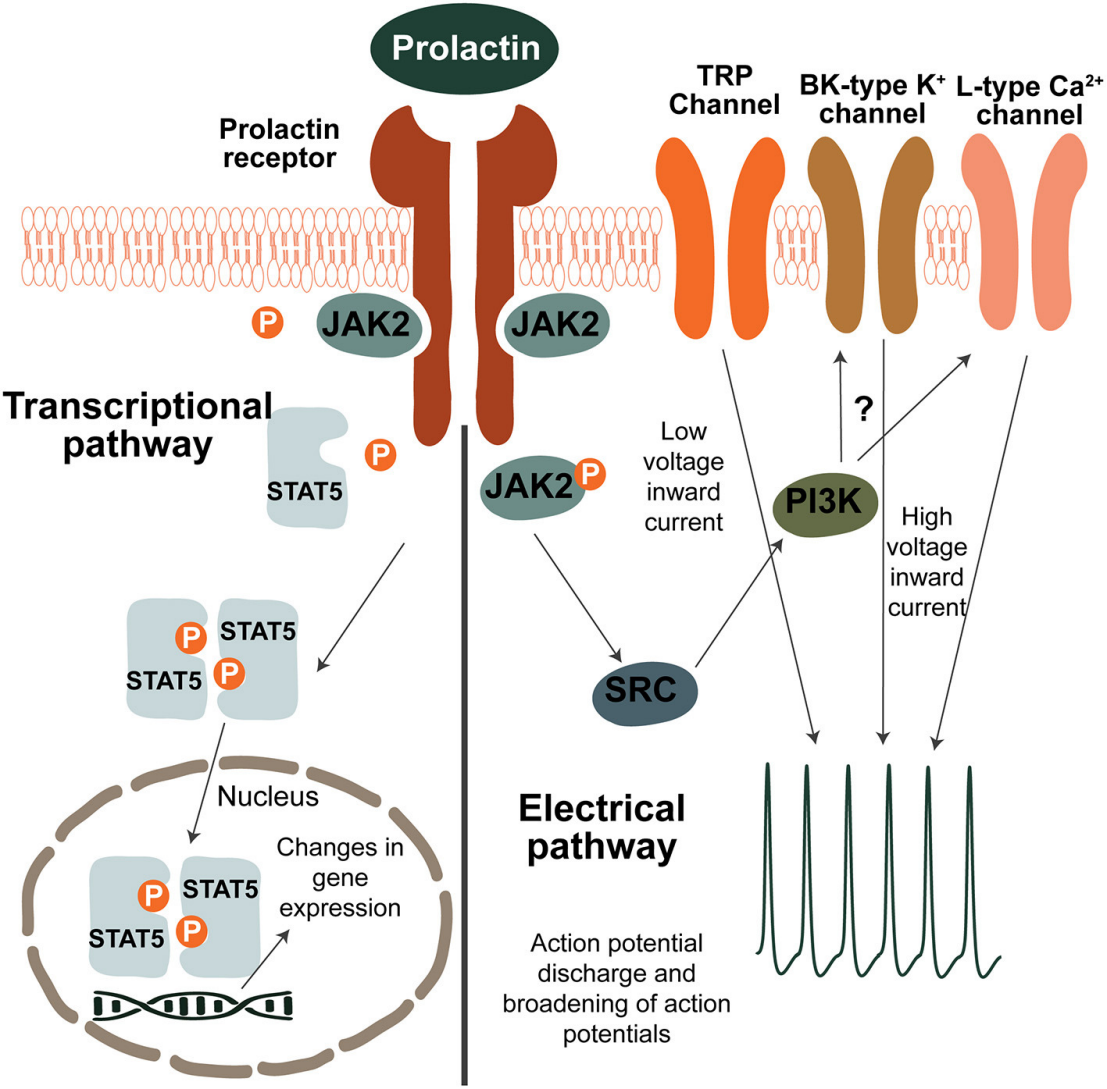
Prolactin-induced signaling pathways in neurons. Transcriptional pathway: binding of prolactin to the prolactin receptor (Prlr) results in activation of JAK2 which in turn phosphorylates several tyroside residues on the Prlr. This causes binding and phosphorylation of STAT5 which then dissociates from the receptor. Two pSTAT5 molecules dimerise and translocate to the nucleus where they lead to changes in gene expression by binging to the promoters of target genes. To induce changes in electrical activity, prolactin activates a low voltage and a high voltage current. The low voltage component is dependent on TRP channels. The high voltage component requires BK-type K^+^ and L-type Ca^2+^ channels and is dependent on activation of PI3K, however the precise mechanism is unknown. It should be noted that although possible pathways of prolactin-induced signaling are illustrated here, prolactin preferentially acts through the different pathways in a cell-specific and reproductive-state specific manner.

The Prlr is part of the class 1 cytokine receptor superfamily and is composed of the prolactin-binding extracellular domain, a 24 amino acid long transmembrane domain and of the intracellular domain (39). The receptor does not have intrinsic tyrosine kinase activity but is associated with the Janus kinase 2 (JAK2) kinase (39, 40). As shown in Figure 1, prolactin binding to the extracellular domain results in dimerization of the receptor and activation of JAK2 which in turn triggers a signaling cascade resulting in phosphorylation and translocation of signal transducer and activator of transcription 5 (pSTAT5) to the nucleus where it exerts targeted effects on gene transcription (39). There are two homologous isoforms of STAT5; STAT5a and STAT5b (11, 17), which are often co-expressed in tissues (10). Although in some tissues, such as the mammary gland, STAT5a predominantly mediates prolactin receptor signaling (41, 42), in the hypothalamus of the brain, where the Prlr is highly expressed, STAT5b is critical for prolactin action (43, 44). To detect prolactin-induced activation in the brain, immunostaining for pSTAT5 can detect phosphorylation of both STAT5 isoforms and has been frequently-used to demonstrate prolactin-induced activity in multiple brain regions, including those known to regulate maternal behaviors (45–50).

Apart from its transcriptional effects, prolactin has also been shown to directly influence neuronal activity (Figure 1). The most pronounced effects have been determined within the short-loop feedback regulation of prolactin secretion. Prolactin acts to stimulate the activity of tuberoinfundibular dopamine (TIDA) neurons in the arcuate nucleus of the hypothalamus (ARN), increasing release of dopamine that subsequently inhibits prolactin release from the anterior pituitary gland. A high proportion (>90% in rats and ~80% in mice) of TIDA neurons express the Prlr (51–53), and in response to an acute application of prolactin, TIDA neurons switch from their archetypal phasic firing pattern to tonic firing (54). Indeed, prolactin was able to increase the activity of a high proportion (~70%) of ARN Prlr-expressing cells, as measured by calcium imaging (55). These rapid effects are observed in both males, non-pregnant females and lactating females (54–57). In TIDA neurons, the high voltage component of the prolactin-induced excitation appears to be dependent on the phosphoinositide 3-kinase (PI3K) pathway and requires BK K^+^ channels, whereas the mixed cationic transient receptor potential (TRP) channels underpin the low voltage component (54). Specifically in females, prolactin also sensitizes TRP channels in sensory neurons and contributes to the regulation of pain responses (58–60). Amongst other brain regions important for maternal behaviors, prolactin has also been shown to have acute effects on neurons in the medial preoptic area (MPOA) (55, 61). Compared to the ARN, however, prolactin induces these effects upon a much smaller proportion (~25%) of MPOA cells (55), suggesting that prolactin regulates the cells in this brain region primarily through its transcriptional pathways.

The paraventricular nucleus of the hypothalamus (PVN) has also emerged as a brain region controlling maternal behaviors and hosting Prlr-expressing neurons. In this brain region, acute application of prolactin can inhibit a subset of oxytocin neurons in virgin and pregnant rats, but not in lactating rats (62–64). Conversely, when specifically examining PVN cells that express the Prlr in the mouse, the acute effects of prolactin are heterogenous, encompassing both inhibitions and excitations in males, virgin females and lactating females (55). However, as with the MPOA, these direct effects on neuronal activity are only seen in a subset of Prlr-expressing PVN neurons (~30%) (55), providing evidence that gene transcription is likely to be the primary method of prolactin-induced effects in the PVN. Currently, little is known about the precise mechanism driving prolactin's rapid effects, highlighting the need for further study on this topic.

## Offspring-Directed Behaviors (Retrieval, Licking, Nursing, and Grooming)

**Figure 2:**
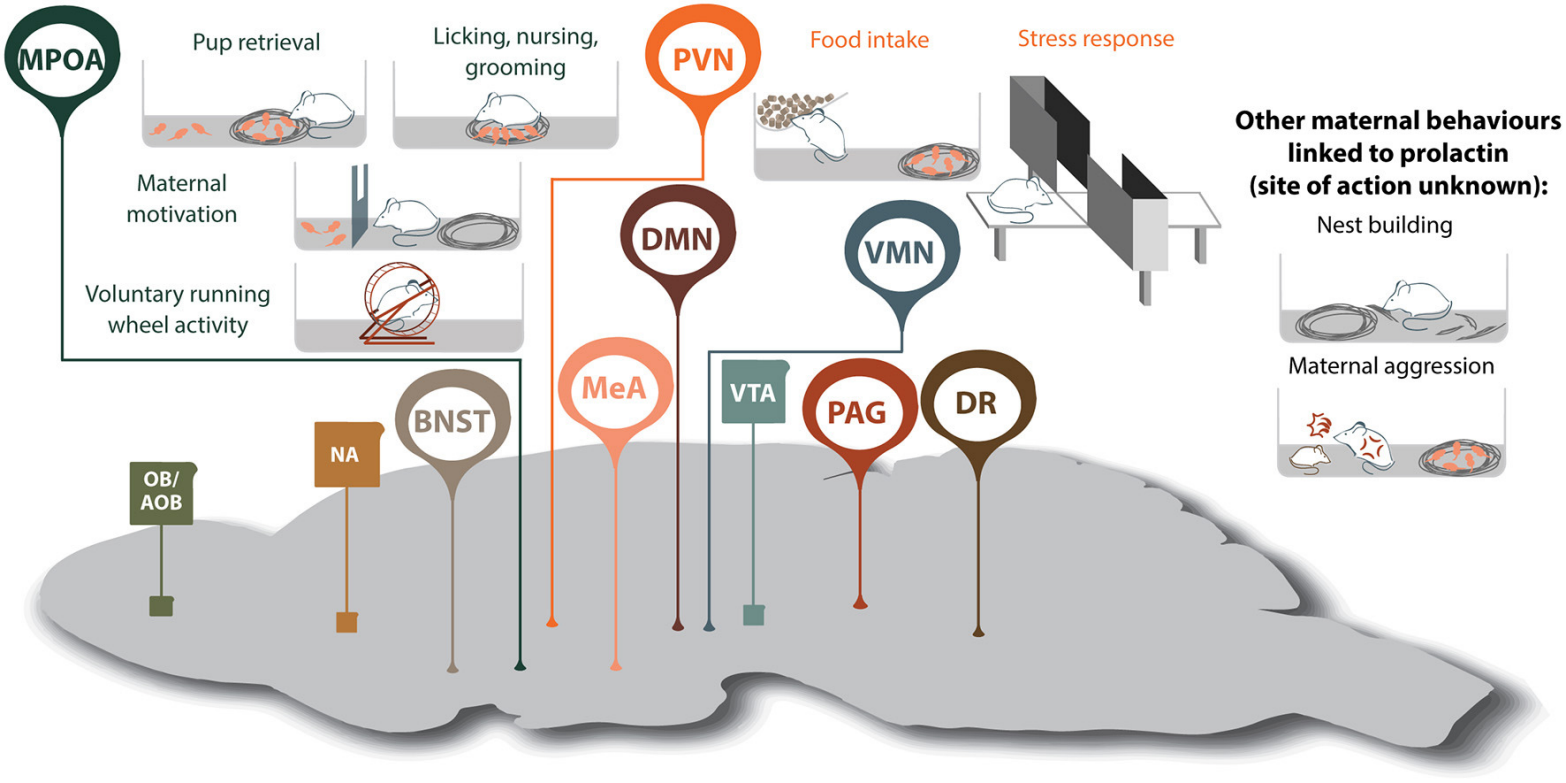
Prolactin-regulated maternal behaviors governed by the maternal neural circuit. Within the neural circuit that regulates maternal behavior, many brain regions show increased prolactin receptor activity during lactation (shown in oval, whereas regions without Prlr but associated with maternal behavior are shown in squares). Note, that although prolactin has been shown to regulate additional aspects of maternal behavior, the brain regions mediating some of these effects are currently unknown. AOB, accessory olfactory bulb; BNST, bed nucleus of the stria terminalis; DMN, dorsomedial nucleus of the hypothalamus; DR, dorsal raphe nucleus; MeA, medial amygdala; MPOA, medial preoptic area; NA, nucleus accumbens; OB, olfactory bulb; PAG, periaqueductal gray; PVN paraventricular nucleus of the hypothalamus; VMN, ventromedial nucleus of the hypothalamus; VTA, ventral tegmental area.

Offspring-directed behaviors promote the well-being of young and include all maternal behaviors that involve direct interactions between a mother and offspring. In rodents, this includes retrieval of offspring, licking and grooming, and nursing/crouching behavior. The first investigation into prolactin's role in this maternal behavior was reported in 1935, where repeated exogenous prolactin administration induced maternal behavior in virgin female rats (65). Subsequent experiments showed that the known pro-maternal hormones, estradiol and progesterone, were insufficient on their own to induce maternal behavior in virgin rats, and highlighted the requirement for pituitary-derived prolactin for this behavior to be elicited (66). Since then, research has sought to understand the types of maternal behavior that lactogenic hormones influence, their site of action, and to identify when during pregnancy and lactation these hormones are important (Table 1 and Figure 2).

**Table 1:** Evidence for prolactin regulating specific aspects of maternal behavior.

**Maternal behavior**	**Reproductive state**	**Prolactin manipulation**	**Effect**	**Species**	**References**
Pup retrieval	Non-pregnant HYPOX/OVX + E/P	Pituitary graft (secreting prolactin)	↑	Rat	(66)
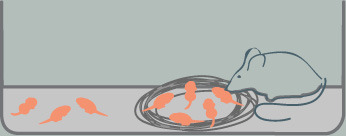	Non-pregnant HYPOX/OVX + E/P	Prolactin (500 μg/ twice daily; 13 days, s.c.)	↑	Rat	(66)
	Non-pregnant OVX+ E/P	Prolactin (400 ng, i.c.v.)	↑	Rat	(67)
	Non-pregnant OVX+ E/P	Prolactin (MPOA; 40 ng per side/twice daily; 2.5 days)	↑	Rat	(67)
	Non-pregnant OVX+ E/P	Placental lactogen (MPOA; 40 ng per side/twice daily; 1.5 days)	↑	Rat	(68)
	Non-pregnant OVX+ E/P	Prolactin receptor antagonist S179D-PRL (MPOA; 0.03 μg/h;7 days osmotic minipump)	↓	Rat	(69)
	Non-pregnant	Prlr KO	↓	Mouse	(70)
	Pregnant	Bromocriptine from day 1—3 of pregnancy (50 μg/day, s.c.)	↓	Mouse	(71)
	Lactating	Heterozygous Prlr KO	↓	Mouse	(70)
	Lactating	Glutamatergic Prlr KO	—	Mouse	(72)
	Lactating	GABAergic Prlr KO	↓	Mouse	(72)
	Lactating	Bromocriptine from day 2—5 of lactation (125 μg/day, s.c.)	↓	Rat	(73)
Litter survival	Lactating	MPOA Prlr KO	↓	Mouse	(72)
	Lactating	Glutamatergic Prlr KO	—	Mouse	(72)
	Lactating	GABAergic Prlr KO	—	Mouse	(72)
Maternal motivation	Lactating	GABAergic Prlr KO (T-maze)	↓	Mouse	(74)
	Lactating	GABAergic Prlr KO (home-cage)	—	Mouse	(74)
Maternal aggression	OVX	Prolactin (100μg, s.c)	↑	Mouse	(75)
	OVX	Prolactin (200μg, s.c)	↑	Mouse	(75)
	OVX	Prolactin (400μg, s.c)	↑	Mouse	(75)
Nest building	Non-pregnant	Prolactin (dry powder, subdermal implant 1.5 mg)	↑	Mouse	(76)
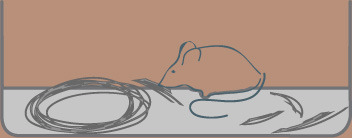	Non-pregnant	Prolactin (hypothalamic, 0.07 mg, prolactin filled tubing)	↑	Mouse	(76)
	Non-pregnant	Prolactin (cortical, 0.07 mg, prolactin filled tubing)	—	Mouse	(76)
	Non-pregnant	Prolactin (subdermal implant, 0.07 mg, prolactin filled tubing)	—	Mouse	(76)
	Pregnant	Bromocriptine from day 26 of pregnancy — parturition (1 mg/kg/day, s.c.)	↓	Rabbit	(77)
	Pregnant	Bromocriptine from day 26 of pregnancy — parturition (1 mg/kg/day, s.c.) + prolactin (5μg i.c.v.)	↑	Rabbit	(78)
	Pregnant + lactating	Bromocriptine from day 26 of pregnancy — day 5 of lactation (1 mg/kg/day, s.c.)	↓	Rabbit	(77)
Stress response	OVX + E	Prolactin (0.01 μg/h; 5 days, osmotic minipump)	↓	Rat	(79)
	OVX + E	Prolactin (0.1 μg/h; 5 days, osmotic minipump)	↓	Rat	(79)
	OVX + E	Prolactin (1 μg/h; 5 days, osmotic minipump)	↓	Rat	(79)
	Lactating	Prlr KO (antisense oligonucleotides, 0.5 μg/0.5 μL/h i.c.v.)	↑	Rat	(80)
Food intake	Non-pregnant	Prolactin (2, 5, 10 μg/μL/h i.c.v.) acute	↑	Rat	(81)
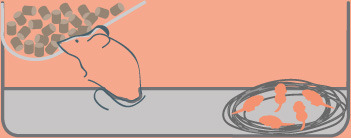	Non-pregnant	Prolactin chronic (14-day osmotic minipumps, 5 μg/0.5 μL/h	—	Rat	(81)
	Non-pregnant	Prolactin chronic (10 days, twice daily 0.3, 1, 3 μg/g s.c.)	↑	Rat	(82)
	Non-pregnant OVX + E	Ectopic pituitary transplants	↑	Rat	(83)
	Non-pregnant	Prolactin chronic (10 days, 800 ng/day, i.c.v.)	—	Rat	(84)
	Non-pregnant	Prolactin chronic (10 days, 800 ng/day, PVN)	↑	Rat	(84)
	Non-pregnant	Prolactin chronic (10 days, 800 ng/day, MPOA)	—	Rat	(84)
	Non-pregnant	Prolactin chronic (10 days, 800 ng/day, VMN)	↑	Rat	(84)
	Non-pregnant	AgRP Prlr KO	—	Mouse	(85)
Voluntary running wheel activity	Non-pregnant	Prolactin (5 mg/kg i.p.)	↓	Mouse	(86)
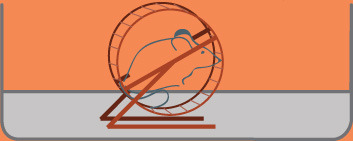	Non-pregnant	Forebrain Prlr KO	↓	Mouse	(86)
	Non-pregnant	Glutamatergic Prlr KO	—	Mouse	(86)
	Pregnant	Forebrain Prlr KO	↑	Mouse	(86)
	Pregnant	GABAergic Prlr KO	↑	Mouse	(86)
	Pregnant	MPOA Prlr KO	↑	Mouse	(86)

*Green shaded rows refer to the role of prolactin on offspring-directed maternal behaviors, while orange shaded boxes describe the role of prolactin on offspring-related behaviors. Upward arrows indicate increase in behavior; downward arrows indicate decrease in behavior; dash represents no change in maternal behavior. E, estrogen; HYPOX, hypophysectomised; i.c.v., intracerebroventricular; i.p., intraperitoneal; KO, knockout; MPOA, medial preoptic area; OVX, ovariectomised; P, progesterone; PVN paraventricular nucleus of the hypothalamus; s.c., subcutaneous; VMN, ventromedial nucleus of the hypothalamus*.

The generation of a Prlr knockout mouse enabled a more thorough assessment of prolactin's role in offspring-directed maternal behavior. In the absence of all Prlr signaling, pup retrieval and crouching over the pups was severely disrupted in knockout virgin female mice and heterozygote dams (70). Maternal behavior could not be assessed in the homozygote knockout dams in these studies since prolactin action in the ovary is required for the maintenance of pregnancy in rodents (87, 88). Confirming the brain as the mediator of these effects, central administration of prolactin induced dose-dependent pup retrieval behavior in virgin female rats at doses ineffective when administered peripherally (67). The MPOA of the hypothalamus was identified as a key site in mediating prolactin's role in regulating maternal behavior, with direct infusion of either prolactin or placental lactogen into the MPOA, inducing maternal behavior in non-pregnant rats at a lower dose than required when administered into the lateral ventricles (67, 68). Similarly, MPOA infusion of a Prlr antagonist delayed the display of pup-induced maternal behavior in non-pregnant rats (69). The MPOA is widely acknowledged as the center of the neural network that governs maternal responses (89–91). The Prlr is expressed in high levels in the MPOA (32, 45, 92), and exogenously administered prolactin induces high levels of pSTAT5 immunolabeling in non-pregnant mice (45). Indeed, single cell sequencing identified ~70 different subtypes of neuronal populations in the MPOA, with the Prlr expressed in a large proportion of these, suggesting prolactin may have a broad role in regulating the MPOA (93). Consistent with elevated levels of placental lactogen during pregnancy, pSTAT5 immunolabeling is also high during late pregnancy in mice (46, 94), and endogenously high circulating prolactin maintains this increased pSTAT5 labeling during lactation in mouse dams (49). While these studies demonstrated that Prlr signaling in the MPOA could influence maternal behavior, it is only recently that conditional knockout studies have revealed that this signaling is essential for normal postpartum maternal behavior. Targeted deletion of the Prlr from the MPOA of adult female mice abolished maternal behavior, with dams abandoning their offspring shortly after parturition (72). Interestingly, conditional deletion of the Prlr (using a Prlr flox mouse) from either glutamatergic neurons (using a VGlut2-Cre mice to target glutamatergic neurons) or GABA neurons (using a VGat-Cre mouse to target GABA neurons) or from both populations failed to have any effect on litter survival or on pup retrieval in the home cage (72). This suggests that the few remaining neurons within this circuit that still express the Prlr (72) are sufficient to enable maternal behavior to be expressed. The MPOA is characterized by a high degree of interconnectivity (95), and conceptually, prolactin signaling on a few cells could be transmitted more broadly throughout the MPOA through this robust network of neurons.

As Prlrs are required for the onset of maternal nursing behavior, important questions can be raised as to the temporal and mechanistic nature of lactogenic activity during pregnancy and lactation. In both rats and mice, pregnancy has been shown to be accompanied by a period of increased neurogenesis (96, 97) which is important for attenuation of the stress response and normal maternal responses in a stressful environment (71). Prolactin secretion during early pregnancy appears to be an important mediator of this increased neurogenesis with reduction of prolactin secretion in early pregnancy, reducing neurogenesis and leading to increased anxiety behavior and poor retrieval behavior of mice in a stressful environment (71). The technical difficulties of manipulating placental lactogen secretion during pregnancy without adversely effecting pregnancy outcomes, represents a substantial challenge in determining the contribution of placental lactogen in regulating maternal behavior.

### Maternal Reward and Motivation

Motherhood is accompanied by high levels of maternal motivation, defined as an increased responsiveness to offspring-related stimuli and a strong drive to seek out interactions with offspring (89, 98, 99). This maternal motivation is essential for the sustained expression of maternal behaviors, especially *in situations* where increased effort is required to provide appropriate care. Various rodent studies have shown that offspring-related stimuli are strongly rewarding to females during lactation, which reinforces their motivation to interact with offspring (100, 101). In contrast, the rewarding value of pups and the level of maternal motivation are normally low in non-lactating rat and mouse females (74, 101). The rewarding effect of pups is dependent on signaling within the reward system in the brain. Transient inhibition of the ventral tegmental area (VTA) by local administration of bupivacaine disrupts the ability of postpartum rats to form preferences for pup-associated contexts (102). This pup preference is also blocked by bupivacaine-mediated inhibition of the MPOA (103), with neuronal projections linking the MPOA to the VTA, and this pathway specifically driving motivated maternal behaviors such as pup approach and retrieval in mice (104, 105).

Studies that specifically focus on the role of lactogenic hormones in maternal motivation have recently started to emerge. A first study reported correlational findings of a link between high maternal motivation in a barrier climbing test and increased Prlr signaling (as measured by pSTAT5 immunoreactivity) in several brain regions, including the MPOA, MeA, PVN, and posterior intralaminar thalamic nucleus (PIL) (47). Increased pSTAT5 immunoreactivity was also found in the MPOA and PIL of pup-experienced virgins that showed increased maternal motivation in this test (47). Recently we have shown a causal relationship between lactogenic action in the brain and maternal motivation. Lactating mice with a conditional deletion of Prlr from GABAergic neurons (using a VGat-Cre mouse to target GABA neurons) showed impaired pup retrieval behavior in a novel T-maze test, without changes in general anxiety levels. Interestingly, maternal motivation of these females was not impaired in a barrier climbing test, indicating that lactogenic action on GABA neurons is necessary for full maternal motivation under specific conditions (74). Given the importance of the MPOA-VTA circuit in directing maternal motivation and the presence of a prolactin-sensitive MPOA-VTA projection (72), prolactin could be acting directly on GABAergic neurons in the MPOA to alter maternal motivation.

Identifying the neural circuits and hormonal factors that promote offspring-directed behaviors in mothers has important implications for the regulation of postpartum mood in women. Postpartum depression affects numerous maternal caregiving behaviors and is accompanied by an increased risk for poor mother-baby attachment (106, 107). Neural imaging studies in postpartum women have shown activation of reward regions of the brain, including the VTA, when women view images of their own infants smiling faces (108–110). In contrast, women with postpartum depression show reduced responses to viewing their own infant's smiling faces, and to hearing their own infant cry (111–113). With an accumulating evidence base for an important role for lactogenic hormones in promoting mother-infant interactions, future investigation into the correlation between impaired lactogenic signaling (to encompass all lactogenic hormones) and the occurrence of postpartum mood disorders in women is warranted.

## Offspring-Directed Behaviors

### Maternal Aggression

Maternal aggression is typically exhibited by postpartum females, but not by non-pregnant females, and allows a mother to guard her young from dangers and perceived threats. Lactating females will therefore engage in both offensive and defensive behaviors toward an unfamiliar intruder (114). This maternal adaptation is an evolutionarily-conserved trait that is broadly exhibited by many different species, both in domestic animals and in the wild and includes rodents, non-human primates and humans (115–122). The specific expression of this aggressive behavior during early lactation occurs at a time when the levels of prolactin are high, suggesting prolactin might be involved in regulating this behavior. Interestingly, in humans, the level of postpartum aggression is higher in women who are breast-feeding compared to formula feeders and nulliparous females (122). Furthermore, lactating or non-pregnant women with hyperprolactinemia (chronically elevated prolactin) display similar levels of heightened hostility compared to control non-pregnant women (123). These experiments assessed aggression using a Kellner Symptom Questionnaire, which provided values of anxiety, depression, somatic symptoms, and hostility (123). Depression and anxiety scores were higher in hyperprolactinaemic patients than postpartum women and controls, yet the hostility levels were similar between the former two groups (123). A more recent study utilized the 90-item symptom checklist to examine similar parameters in hyperprolactinaemic patients (124). In contrast to earlier studies, anxiety and depression parameters were not different between control group and hyperprolactinaemic patients (124). However, hostility levels were higher in hyperprolactinaemic patients (124), again indicating that prolactin might play in role in regulating aggressive behaviors. It is important to note that a vast majority of these studies examined subjects whose prolactin levels were abnormally high due to a medical condition, thereby not excluding the effects could be attributed to other associated comorbidities.

Animal models have been used to interrogate the neural circuitry underlying aggressive behavior in both males and females, with the medial amygdala (MeA) and ventromedial nucleus of the hypothalamus (VMN) emerging as key regulatory sites (125, 126). An initial indication that prolactin might be involved in maternal aggression came from a study carried out on golden hamsters (118). The level of aggression displayed by these animals was highest during pregnancy and lactation, and lowest during pseudopregnancy (development of signs of pregnancy without the presence of an implanted embryo) and estrous (118), matching the patterns of blood prolactin levels in this species. In ovariectomized white-footed mice (Peromyscus leucopus), prolactin administration increased levels of intruder-directed aggression in a dose-dependent manner (75). At the higher prolactin doses (>200 ug), levels of aggression were similar between prolactin-injected ovariectomised mice and lactating females (75). Surprisingly, drastically reducing prolactin levels through hypophysectomy (removal of the pituitary gland), did not affect aggression in parturient Rockland-swiss mice (127). However, this approach would not suppress Prlr signaling during pregnancy, as placental lactogen secretion (the primary ligand for the Prlr during pregnancy) would not be affected by hypophysectomy. Interestingly, Prlr signaling in both the MeA and VMN is increased in during pregnancy and lactation (49, 94), suggesting lactogenic action in these regions may modulate maternal aggression at these times.

In humans, maternal aggression is an important component of postpartum mood, and disturbances in the regulation of this adaptation can lead to heightened anger toward self, the child or other family members (128). The likelihood of displaying abnormal levels of anger is also associated with postpartum depression and lower emotional availability for their children (128). Similarly, elevated postpartum anxiety is linked with abnormally high levels of intruder-directed maternal aggression (129), an indicator of hypervigilant parenting. Understanding how maternal protective behaviors are controlled in a healthy pregnancy and lactation is therefore key for developing effective strategies to promote development of healthy mother-child interactions.

### Nest Building

An important component of maternal behavior, particularly in altricial species, is maternal nest building. This energetically-costly process ensures offspring survivability as the young from these species have limited capacity for thermoregulation and locomotion. As with other maternal behaviors, nest building is highly conserved between species.

Female mice can construct either a sleeping nest or a brood nest, the latter being considered the maternal nest (130). The brood nest is significantly larger and encloses the animals so that they cannot be easily seen while in the nest. Mice begin to build the brood nest during early gestation, with this behavior enhanced by exposure to pups and able to be blocked by olfactory bulb removal (130, 131). Pseudopregnant mice also build maternal nests compared to virgin controls, suggesting a hormonal basis for the regulation of this behavior (132). Evidence of prolactin's involvement in maternal nest building came from early studies administering prolactin to virgin mice (76). Virgin female mice that received either hypothalamic or subdermal implants of prolactin built superior nests than control mice or mice with cortical-implants of prolactin (76). Manipulation of prolactin levels in rabbits are also able to alter maternal nest building (77, 78). For example, blocking prolactin secretion from day 26 of pregnancy until parturition *via* sub-cutaneous bromocriptine injections significantly reduced the construction of maternal nests in rabbits (77). This deficit in maternal nest building was rescued in rabbits receiving intracerebroventricular (i.c.v.) prolactin, suggesting a direct role of this hormone in the execution of this behavior (78, 133). Several studies have indicated that MPOA neurons drive nest building (134, 135), and with Prlr expression in this brain region regulating other maternal behaviors (72), the MPOA could conceptually also mediate the effects of prolactin on nest building.

The equivalent of nesting behaviors in human mothers, may be preparation of the physical environment and social selectivity, both of which peak in the third trimester of pregnancy (136). Interestingly, this corresponds with peak secretion of placental lactogen, and suggests the lactogenic hormones may have a causal role in this behavior. However, further studies are required to determine the precise mechanism underlying hormonal regulation of nest building behavior, and whether it may be relevant to understanding forms of maternal neglect in humans.

### Attenuated Stress Response

During pregnancy and lactation, the reactivity of the hypothalamic-pituitary-adrenal (HPA) axis of multiple species, including humans, is drastically reduced (137, 138). Stress during pregnancy and lactation can detrimentally impact pregnancy outcomes, increasing the likelihood that the mother will abandon her pups and the prevalence of postpartum mood disorders (139–141). Gestational stress also has negative effects on fetal development and contributes to programming hyperactive stress responses in the offspring (140, 142–144). Reduced HPA axis reactivity is therefore a critical adaptation that serves to protect both the mother and offspring from these stress-related effects. In mice, suppression of the stress response during lactation is evident both following a restraint or endotoxin stressors, with attenuated adrenocorticotropic hormone (ACTH) and corticosterone secretion compared to virgin females (145). Similarly, in humans, temperature or exercise-induced stressors are dampened during pregnancy and lactation, respectively (137, 146).

As a potential regulator of these changes, prolactin has been shown to have dose-dependent anxiolytic effects in non-pregnant rats (79, 147). Chronic administration of prolactin for 5 days in virgin rats reduces ACTH and corticosterone secretion following a restraint or anxiety-like stressor (79). Interestingly, following exposure to the stressor, prolactin-injected animals showed reduced c-fos immunoreactivity (a marker of neuronal activation) in the PVN (79), a key region controlling the stress response, hinting at a mechanism by which prolactin exerts its anxiolytic effects. The PVN is host to corticotropin-releasing hormone (CRH) neurons, a population proven to regulate a plethora of physiological and behavioral stress-related responses (148–150). Evidence from earlier studies suggested prolactin can regulate the activity of CRH neurons in hypothalamic explants (151). More recently, studies in mice have demonstrated that PVN *Crh* mRNA levels are lower during pregnancy and lactation compared to the virgin controls (152). Pup-removal, and thereby blocking suckling-induced prolactin secretion, lead to an increase in PVN *Crh* mRNA and this was restored to the low lactating levels following prolactin administration for 24 h in mice (152). However, these effects do not appear to be direct as *Prlr* mRNA is not expressed by PVN CRH neurons (153). Similarly, both in virgin and lactating mice and rats, prolactin does not directly activate PVN CRH neurons (as measured by pSTAT5 immunoreactivity) (153). Nonetheless, downregulation of Prlr in the brain resulted in increased anxiety-related behaviors and higher ACTH secretion in lactating rats (80). Thus, while the precise brain region where prolactin acts to exert its anxiolytic effects is not yet known, it is evident this pituitary hormone plays an important role in regulating this maternal adaption.

In women, increased anxiety and stress during pregnancy are risk factors for preterm birth and low birth weight while also negatively impacting mother-child interactions (154–156). Similarly, the high prevalence rates of perinatal anxiety in women (157, 158) highlights the importance of understanding how stress is regulated during pregnancy, particularly since these early life interactions are critically important in child development.

### Metabolic Adaptations

Profound changes in metabolic regulation are essential to provide sufficient energy for the development of the growing fetus and to meet the high metabolic demands of lactation. From a behavioral perspective, this involves changes to both food intake and voluntary energy expenditure. Food intake drastically increases during pregnancy and lactation in humans and other mammalian species (159–163). Interestingly, lower body weight increase during pregnancy is associated with litter loss and overall poor pregnancy outcomes (164). Research on the neuronal pathways regulating food intake and energy balance has largely focused on the medial basal hypothalamus, with the ARN considered to be the center of the neural circuit regulating food intake. This brain structure is host to two key neuronal populations known to affect energy balance; the adjacent yet functionally antagonistic, proopiomelanocortin (POMC) and agouti-related peptide/neuropeptide Y (AgRP/NPY) neurons that exert powerful effects on satiety and hunger, respectively (165–167). In order to exert effects on feeding behavior, some of the important hypothalamic projection regions of these ARN neuronal populations include the PVN, dorsomedial hypothalamus, ventromedial hypothalamus and lateral hypothalamus (168–170).

Multiple studies have linked prolactin action with food intake and body weight in non-pregnant rats (summarized in Table 1) (81–83). Prolactin appears to act at the level of the PVN to influence appetite, with chronic prolactin administration in this brain region increasing food intake (84). In the pregnant state, rodents eat more and become insensitive to the effects of the satiety hormone, leptin, particularly in the hypothalamic regions associated with leptin's effects on food intake (159, 171). This leptin resistance appears to be driven by placental lactogen secretion in the second half of pregnancy, with prolactin administration to pseudopregnant rats blocking the satiety effects of leptin (172). During lactation, increased food intake is necessary to meet the high energy demand of milk production. This hyperphagia appears to be dependent on increases in the orexigenic AgRP and NPY, and a decrease in POMC expression (173–175). There is evidence suggesting prolactin plays a role in regulating the hyperphagia of lactation, but not through direct actions on POMC or AgRP neurons in the ARN (85, 176). A population of NPY neurons in the DMN are also important for regulating energy metabolism and food intake (177, 178), with many of these showing increased prolactin receptor activity during lactation compared to the non-pregnant state (33, 49, 179). Expression of NPY mRNA in the DMN increases during lactation and blocking prolactin secretion (*via* bromocriptine administration) abrogates this effect (179), suggesting that prolactin stimulates production of orexigenic NPY during lactation. To further evaluate the precise role of DMN Prlr-expressing cells in mediating lactational increase in food intake, future studies would be required to precisely knockout Prlr in this brain region.

In order to adapt to the huge energy demands of pregnancy and lactation, changes in voluntary activity accompany increased food intake. Even from very early in pregnancy, rodents conserve energy by reducing voluntary running wheel activity (164). Interestingly, one study has shown that this reduction in running behavior can predict lactation success in mice (164). Mouse dams who abandoned their litters showed greater running wheel activity and lower weight gain during pregnancy compared to the pregnancies of dams who successfully reared their litters (164). Recent data suggests Prlr activity plays an important role in regulating this voluntary running behavior. In non-pregnant females but not males, peripheral prolactin administration can acutely reduce running wheel activity without affecting general locomotion (86). Furthermore, forebrain-specific knockout of Prlrs (using a Cam kinase II Cre mouse to target forebrain neurons) blocks the pregnancy-induced decrease in running wheel activity, with the MPOA and GABA neurons likely to be mediating these effects of prolactin (86).

The reduction in physical activity during pregnancy is also apparent in human mothers (180). Despite positive attitudes toward physical exercise, there is a significant decrease in physical activity engagement during pregnancy (180). Since increased weight gain during pregnancy is associated with heighted risk of complications and lower delivery outcomes (181), understanding maternal metabolic adaptions is imperative. Although the rodent studies have provided evidence for lactogenic hormones playing an important role in regulating maternal metabolic adaptations, the precise mechanisms mediating these effects are currently unknown, and warrant further investigation.

## Transgenerational Effects

A growing field of research has demonstrated behavioral transmission of postpartum behavior from mothers to female offspring. Most commonly, this has been used to investigate the effect of stress on subsequent offspring's maternal behavior. In these studies, female rat pups that were separated from their mothers for either short or long periods of time, went on to exhibit impaired maternal behavior with their own offspring (182, 183). In addition to direct effects on maternal behavior, prolactin appears to have a transgenerational effect on maternal behavior. Reducing prolactin content of milk in lactating rat dams decreases maternal responses to foster pups in the virgin adult female offspring (73). Mice with a deletion of the Cbl-interacting protein of 85 kDa (Cin85) were able to nurture and raise pups, however, when producing their own pups, these F_1_ offspring were found to have severely disrupted maternal care (184). Examination of prolactin secretion in the first generation of Cin85-deleted mice, showed greatly reduced prolactin production during late pregnancy and early lactation, and prolactin administration to these mice during pregnancy was able to rescue the subsequent offspring maternal behavior (184). This indicates that the *in utero* prolactin environment can alter maternal behavior of offspring through non-genomic transmission. Studies looking at transgenerational effects of high and low licking and grooming rat mothers, have found changes in the methylation of the estrogen receptor alpha promoter in the MPOA, which may alter estrogen sensitivity in this region and account for altered maternal responses of offspring (185). Although the mechanism underlying a transgenerational effect of prolactin is unknown, it could involve a similar epigenetic mechanism to alter sensitivity to prolactin in key brain regions that regulate maternal behavior.

## Lactogenic Hormones Amongst the Changing Hormonal Profile of Pregnancy and Lactation

Although this review focuses on the role of lactogenic hormones on the multiple facets of maternal behavior, it is important to note that multiple hormones and factors influence maternal behavior. Indeed, lactogenic hormones are not acting in isolation, and there is considerable overlap and interactions between these regulators of maternal behavior. Briefly discussed here are some of the key hormonal regulators of maternal behavior that are known to interact with lactogenic hormones.

One significant convergence point for some of these factors is the interaction with TIDA neurons in the ARN, where stimulation (e.g., by thyrotropin-releasing hormone, orexin, oxytocin) or suppression (e.g., by serotonin/5-HT, estrogen, enkephalin, dynorphin) of TIDA neuronal activity, can alter secretion of prolactin from the anterior pituitary (19, 186–193), and therefore has the potential to also indirectly influence maternal behavior. For example orexin inhibits the activity of ARN TIDA neurons resulting in increased prolactin secretion, and is known to alter maternal behavior in a dose dependent manner (194, 195). In postpartum mice, high i.c.v. doses (0.3 μg) of orexin worsened pup retrieval and decreased nursing and nesting behavior, whereas at lower doses (0.06–0.1 μg), orexin mildly improved pup licking and grooming (194). The MPOA appears to mediate some of these effects with MPOA administration of a orexin receptor antagonist increasing pup nursing time in rat dams (196). However, given the effect of orexin in TIDA neuronal activity, it is possible orexin could also indirectly be influencing maternal behavior through modulating prolactin secretion.

Serotonin (5-HT) is also known to promote prolactin release (188, 197), and 5-HT neurons can regulate maternal behavior. Deletion of 5-HT neurons from the brain impairs behaviors such as pup retrieval, nest building, and nursing, without affecting maternal protective behaviors (198). Specifically, 5-HT neurons located in the largest raphe nuclei, the dorsal raphe, are important for pup retrieval and survival (199). This was also associated with reduced pup milk intake, suggesting reduced prolactin inhibitory tone (199). Interestingly, evidence from an earlier study could provide a mechanistic explanation for this phenomenon as dorsal and median raphe lesions reduced suckling-induced prolactin release (200). Due to the wide use of selective serotonin reuptake inhibitors to treat maternal mood disorders, it is important to consider the changes of 5-HT during the postpartum period and how these impact maternal behaviors (201).

The role of oxytocin in stimulating maternal behavior has been the subject of much research. Acting as a neurotransmitter, oxytocin is also released centrally by neurons in the PVN and supraoptic (SON) nuclei and can alter social and maternal behaviors. Central oxytocin administration to ovariectomised and estrogen primed rats can induce maternal behaviors such as grooming, nest building and pup retrieval (202). In particular, retrieval in response to pup calls is dependent on oxytocin receptors expressed by left auditory cortex neurons (203). Oxytocin is also known to influence maternal aggression in postpartum females (114) and in homozygous oxytocin knockout mice, the duration of time spent attacking the intruder was significantly reduced (204). In rats, Prlrs are expressed on oxytocin neurons, and in virgin and pregnant rats, i.c.v prolactin administration inhibits the activity of these neurons (62–64, 205). Interestingly this inhibition is lost during lactation (63), potentially enabling both these pro-maternal factors to be released at higher levels.

The establishment of sex steroids playing a key role in regulating maternal behavior dates back to the 1970's, whereby nulliparous ovariectomised females treated with estrogen retrieved pups significantly faster than the control rats (1, 206). The MPOA again emerged as the brain region important for mediating estrogen's effects on pup retrieval, with knockdown of MPOA estrogen receptor α (ERα) expression leading to decreased pup nursing, licking, and retrieval (207). Estrogen could also indirectly promote maternal behavior through regulating lactogenic hormone signaling, with estrogen treatment upregulating Prlr mRNA expression in the brain (208, 209). Progesterone has emerged as a negative regulator of maternal behavior and primarily influences maternal behavior through modulating the actions of other hormones (210). In ovariectomised, nulliparous rats, chronic progesterone administration suppressed Prlr mRNA expression in the MPOA (211). Progesterone withdrawal as seen in rodents and functional withdrawal of progesterone as seen in women at the end of pregnancy (212), may thus contribute to facilitating the stimulation of maternal behavior by lactogenic hormones.

## Conclusions and Future Directions

Motherhood is a period of dramatic physiological and behavioral change that ultimately ensures healthy development of offspring. While some studies have studied the mechanisms driving maternal behaviors in humans, rodent models have proven invaluable in deciphering how these adaptations are achieved. In both humans and rodents, lactogenic hormones are elevated during pregnancy and lactation, and by acting on the widely-distributed Prlr, regulate multiple behavioral functions (10, 213). Amongst these, we have highlighted that these lactogenic hormones exert profound effects on a diverse range of both young-directed and young-related maternal behaviors. There is substantial evidence that Prlr signaling is important for offspring-directed behaviors including pup retrieval, licking nursing, grooming and on increasing maternal motivation, as well as offspring-related behaviors such as reducing anxiety, building a nest and maternal aggression (Figure 2). For these behaviors, prolactin actions have been linked, at least in part, through actions on the MPOA and PVN. Interestingly, Prlr signaling is also increased in multiple additional regions of the maternal brain that are known to be important for governing aspects of maternal behavior, such as the MeA, and yet, the role of prolactin in these regions is unclear (Figure 2). With such broad implication for regulating mood and behavior in mother, there is clearly a need to more precisely understand the neuronal circuits that govern these prolactin-driven maternal adaptations.

Alongside this period of extensive maternal adaptation in pregnancy and lactation, is also an increased risk of developing mood disorders. Maternal mood disorders represent a significant health burden worldwide with ~20% of women suffering either from postpartum depression or postpartum anxiety. Maternal mental health problems during pregnancy and the postpartum period represent a significant barrier in developing healthy mother-infant interactions. Treatment for postpartum mood disorders often relies on either psychological intervention such as cognitive behavioral therapy or pharmacological intervention including antidepressants or hormone therapy. However, until we have a better understanding of the physiological processes that lead to both normal and abnormal adaptations to pregnancy, and the molecular mechanisms that underlie these processes, our ability to develop new therapies to treat impaired adaptations during pregnancy and early lactation are limited.

## Author Contributions

TG, JS, and RB wrote the manuscript. TG created the figures. TG, DG, and RB edited the manuscript. All authors contributed to the article and approved the submitted version.

## Funding

This work was supported by a Health Research Council of New Zealand Project Grant and conducted during tenure of the Sir Charles Hercus Health Research Fellowship (by RB) of the Health Research Council of New Zealand.

## Conflict of Interest

The authors declare that the research was conducted in the absence of any commercial or financial relationships that could be construed as a potential conflict of interest.

## Publisher's Note

All claims expressed in this article are solely those of the authors and do not necessarily represent those of their affiliated organizations, or those of the publisher, the editors and the reviewers. Any product that may be evaluated in this article, or claim that may be made by its manufacturer, is not guaranteed or endorsed by the publisher.
